# Cases in Practice

**Published:** 1856-01

**Authors:** J. C. Hinsey

**Affiliations:** Delonega, Iowa


### CASES IN PRACTICE.

BY J. C. HINSEY, M. D., DELONEGA, IOWA.

CASE 1. Called June 26th, 1855, to see John Reece, aged 30 years, nervo-bilious temperament. Has just returned from a tour in Missouri. Has been ailing for several days, with chills and fever. Took two doses of Calomel during the past week, which operated well, but did not feel much better. Had constant and severe pain in his head, especially in the frontal region. Sweats much at night. Has been subject, for two years past, to a severe cramping of the stomach, which returns periodically, say every four weeks, and lasts from one to twelve hours. I had prescribed strychnia for the last three months, with some benefit. Appetite has been variable for several days, attended with a feeling of lassitude, and indisposition to locomotion.

*Present Symptoms.*—His external appearance indicates loss of vitality, skin yellow, evidently tinged with bile, and warmer than natural, also very dry and harsh. Great pain in the head and back, together with a feeling of soreness all over the body. Restless, torpor of the bowels, as also of all the secretions.—Urine scanty and high-colored, depositing a flaky sediment. Tongue heavily coated in the centre with a thick, yellowish brown fur, and very red at tip and sides. Gums red and presenting a scurvy-like appearance. Breath smells feverish, respiration somewhat hurried, but no cough. Puerile respiration slightly marked in the sub-clavicular region of both lungs, but more in the right side. Some tenderness on pressure in the right Iliac region. Pulse 110, quick and irregularly intermitting. Head hot and extremities cold. Sleeps much, but is easily aroused, does not call for much drink.

*Diagnosis.*—Typhoid fever, complicated with thoracic derangement.

*Prognosis.*—Unfavorable.

*Treatment.*—I began the treatment by prescribing

℞ Sub. Mur. Hydg. grs. x,

Ipecac grs. x,

M. Divide into four powders. Give one every three hours, followed with Ol. Ricini and Ol. Terebinth, equal parts. Warm bathing to the extremities, and cold to the head, with directions to keep the room quiet and well ventilated.

June 27. 9 o'clock, A. M. Patient some better. The medicine has operated both as an emetic and cathartic. The discharges from the stomach of a bilious character, as also those from the bowels. Pulse 90, soft and more regular. Tongue more moist, but the edges rather redder than yesterday. Skin softer and not so dry. Pain in the head and back much less. Kidneys remain inactive, the urine being red and very scanty. Ordered

℞ Sulph. Quinia grs. ij,

Pulv. Doveri grs. ij,

Every three hours, alternated with Spts. Nitre.

6 o'clock, P. M. Patient has sweated a little during the day. Pulse 95, and tolerably full. Passed urine more freely, and it is not so highly colored. Tongue about the same as in the morning. Continued same treatment.

June 28th. 9 o'clock, A. M. There is some indication of improvement in patient's condition. A more perfect remission of fever. Pulse 85 and pretty regular. Tongue looks like cleaning off, but is still too red. Skin cool and soft. Eat a little toast and drank some tea. Treatment same as yesterday.

6 o'clock, P. M. Has had much more fever this afternoon. Head very hot, with a disposition to mental derangement.—Bowels have not moved since yesterday morning. Ordered Pil. Hydg. grs. x.

June 29th. 9 o'clock, A. M. Bowels moved since last night. Fever continued until nearly morning. Tongue dry and slightly swollen; red papillæ on the point and sides. Skin dry and rough. Pulse 100, and rather weak. Pain in the head aggravated by moving. Ordered

℞ Calomel grs. ij,
Ipecac grs. ij,

Every four hours. Constant cold to the head; the general surface sponged when hot and dry.

6 P. M. Pulse 105; skin a little more moist. Pain in the head continues, with slight delirium and stupor. Continue treatment as in the morning.

June 30th. 9 A. M. No change since yesterday. Bowels moved twice. Stupor increased some. Pulse 90. Continue Calomel and Ipecac, with ten drops dil. Sulph. Acid every six hours.

6 P. M. Gums present some symptoms of ptyalism, the mercurial dropped. Pulse 88 and regular, but an increase of the delirium, and irregular action of the muscular system generally. Tongue cleaner and more moist, but still too red. Bowels begin to be a little tympanitic, with tenderness on pressure, and a disposition to frequent discharges. Patient quite weak, not having taken any nourishment. Ordered

℞ Emulsia Gum Acac ℥iij,

Ol. Terebinth ℥j,

Tinct. Opii. ʒji,

M. Teaspoonful every three hours.

July 1st. 9 A. M. Delirium continues, with the pulse only 88, soft and regular. Symptoms of glandular ulceration of bowels more prominent. Continue the Terebinth mixture, with wine whey every three hours.

6 P. M. Bowels moving too frequently, discharging a dark colored offensive liquid. Ordered

℞ Acetate Plumbi grs. jii,
Opii. Pulv. grs. j,

Every four hours.

July 2d. 9 A. M. Patient much the same as yesterday. Stupor continues unabated. Bowels moving too often. Continue the emulsion and the lead powders.

6 P. M. Tongue more moist and not so red. Pulse 85 and rather weak. Tympanitis subsiding a little. The bowels have not moved so often during the day. Has taken some squirrel broth.

July 3d. 9 A. M. No change in the symptoms. Continue same treatment, with ice to swallow and applied to the head.

6 P. M. Patient about the same; gave Sulph. Morphia gr. j, and continue as before.

July 4th. 9 A. M. Less heat of skin. Tongue more moist. Bowels quiet. Pulse 85. Cannot take the emulsion. Has occasional vomiting of a bilious and muco-purulent matter, which is very offensive. Ordered nothing but iced wine and water.

4 P. M. Patient is still vomiting the same kind of matter. Gave Sulph. Morphia gr. j. Bowels moved but once since yesterday.

July 5th. 9 A. M. Still vomiting, but seems a little better otherwise. Pulse 85 and slightly irregular. Ordered Muriatic Acid, three drops every three hours.

12 M. Saw the patient in company with Dr. Taylor, of Ottumwa. Pulse 85 weak and intermitted. Tongue red and dry. Vomiting still continues, but less quantities thrown up. The matter ejected is more bilious, with less of the muco-purulent admixture. Bowels quiet. Less stupor, the eye not so much injected, and they are more natural in appearance. Respiration natural and easy; skin cool; thirst not so great. Continue the acid. Wine whey and emulsion of Turpentines. Give as nourishing diet as can be bourne.

6 P. M. Is slightly better, being more calm, and free from pain; is more sensible of his situation. Pulse 85. Continue treatment as above, with half grain Morphia, if he does not rest well to-night.

July 6th. 9 A. M. Heavy stupor. Bowels not moved since day before yesterday. Pulse 88 and weak. Vomits occasionally, the matter thrown up more bilious; tongue red and dry. Ordered an enema of salt and water, and continue wine as before.

12 M. Enema has operated, bringing away a large quantity of mucus membrane in shreds, and a small quantity of fœces of a yellowish color. Continue wine, and gave pill every four hours of Pil. Hyd. grs. jii and Cal. gr. j.

July 7th. 9 A M. Patient continues nearly the same. Still vomits. Bowels moved twice during the night. In addition to the treatment, ordered cold decoction of peach leaves.

6 P M. Patient is better than he has been for several days. Pulse 78, regular and soft. Bowels quiet; vomits much less; tongue not so red and more moist. Mind clear, and not so much sub-sultus tendium. Skin more natural, and better color. Urine more freely secreted. Give nothing but wine to-night.

July 8th. 10 A M. Patient is a great deal better this morning. Pulse 77, slow and soft;—tongue not so red, the papillæ disappearing; has not vomited since last night; slept easy and natural; bowels moved this morning, the discharge being more natural in appearance and consistence. Passed water freely; feels much weaker than at any previous time; mind is perfectly calm; has drank a cup of tea, and eaten a little chicken, which he has not thrown up. Discontinued everything but the wine.

5 P M. Bowels have been discharging frequently a thin, fetid, dark-colored and bloody matter. Pulse 85 and weaker than this morning. Tongue more red and dry. Gave Sub. Mur. Hyd. grs. ji, Acetas Plumbi grs. iv to check the discharges.

July 9th. 8 A M. Bowels moved three times during the night; pulse 82 and very weak; tongue same; passed large quantities of urine through the night; skin looks and feels better, is not so comatose. Wine whey and emulsion as before.

2 P M. Much the same as in the morning; pulse 86 and weak; bowels moved twice since last visit; tongue less dry, and not so red; scarcely any pain on pressure on the abdomen. Has taken some gruel to-day. Ordered

℞ Sulph. Quinia grs. ji,
Pulv. Doveri grs. ji. Every three hours.

8 P M. Patient much better than at any time before; skin moist and cool; tongue more moist, and not so red; the fissures are disappearing; bowels moved twice this afternoon, there being more consistence in the discharges; stomach quiet; singultus—which has existed for several days—is subsiding. Continue the wine whey, and the lead to regulate the bowels.

July 10th. 8 A M. Patient still better—more rational. Continue wine and Muriatic acid alternately.

July 11th. 8 A M. Rested well last night; had two discharges from bowels, fetid and watery. Acid and wine continued, with nourishing diet.

July 13th. 9 P M. Improving slowly. Has continued the acid and wine at regular intervals.

July 14th. Has been worse to-day; vomited green bilious matter; drowsy; discharges of bloody urine; pulse 85, and irregular; tongue dry, red and perfectly clean—glassy in appearance. Hydg. Cum Creta grs. ji, Sulph. Quinia gr. j every four hours, in addition to the vinous stimulant.

July 16th. Is improving slowly under the nourishing plan of treatment, with an occasional dose of the acid.

July 18th. Patient sat up this morning while the nurse made his bed; all his symptoms are of returning health. Wine, acid, and nutritious diet are now his only reliance.

July 22d. Has been convalescing very rapidly, until to-day he has had a severe fit of ague. Ordered Sulph. Quinia grs. jii, every two hours, until four doses are taken.

July 25th. Has had no chill since last report; is doing remarkably well in my opinion.

July 28th. Consider my patient able to take care of himself, so I discharged him to-day.

I report the above as an example of the treatment pursued by me in a great majority of cases of Typhoid fever, and I have been so well satisfied with the result—having lost only one case in twenty-six—that I do not feel disposed to change it, unless I am well convinced that there is a more judicious and safe mode of treatment. I would very much like to hear from the various localities in the State, where this fever is prevalent, in regard to the mode of treatment adopted by the physicians practising in the same.

CASE 2. Called this morning, Aug. 10th, at 7 o'clock, to see Miss ———, aged 19, who has just been taken violently ill.—Has not been very well for a year or two—subject to repeated attacks of headache. There has been an entire suppression of the menses for two months or more past. Six days ago she was out in a severe rain storm, and had to remain with her wet clothes on for some time. Since then she has not felt well—complaining of headache, and being sometimes a little feverish. Last night she came home from school, a distance of a mile and a half, and ate her supper as usual; only remarking that she had a queer kind of headache. Her mother, who is quite a sensible doctoress, gave her about ten grs. Calomel and five of Ipecac. At midnight she

(her mother) got up and gave her a dose of salts; at 2 o'clock, the patient herself got up and took down another dose, saying she did not want to be salivated if she could help it. At about 4 o'clock the medicine operated very freely; and also again in an hour afterwards. Patient went to sleep and slept well for an hour or more, when she arose and dressed herself. She had not been up long before she said that she felt too weak to stay up, and would have to lay down. A few minutes after laying down, she was seized with a violent convulsion, which lasted a full half hour. I now found her but slightly convulsed, all her muscles in great nervous excitement, however. Her mother was feeding her Ipecac, which I directed her to continue, with a view to produce emesis. Her pulse was 160 per minute, and fluttering; a great degree of congestion of the brain and lungs. Head very hot, and extremities cold as death. Pupils of her eyes dilated to their fullest extent; the spasms appeared in their nature, more like those produced by strychnia than anything else I could compare them to, and resembling the agitation produced by very powerful interrupted currents of electricity, sent through the system. She was entirely unconscious of anything, but could swallow a little. There was also a strong pulsation, apparently in the uterus. I applied hot flannels to her feet and hands, and strong sinapisms to the inside of her thighs; ether and cold water to the head. Failing to produce emesis with the Ipecac, I gave her three grains of Tart. Ant., but with no better effect.

Afterwards I gave her in succession, Hoffman's Anodyne, Sulph. Ether, Morphia, &c., but with no sensible effect. I also cut off the cephalic vein in the left arm, but could get no blood. The spasms continued to increase in violence in spite of everything that was done for her until 10 o'clock, when she died.

*Query.*—Did this case resemble Puerperal Hysterites, and was the treatment proper under the circumstances?

REMARKS.—The example of the author of the above cases should be followed by others in the preservation of the particular facts of each case, as presented from day to day. Although these cases may be much abbreviated generally when presented to the public, yet, it is of the utmost importance to preserve the record for future reference and for statistics. We have, in this instance, published the history as given at length that others may be able to compare symptoms as well as note the remedial measures adopted from time to time.

One common error in the reports of cases is to give symptoms and the treatment without a suggestion as to the probable pathological condition at the time, or a hint for the reason for the adoption of a plan of treatment, or the cause which requires a change in that previously pursued. If this objection were but removed we would be better able to understand the opinions of those who may report cases and the reasons for their action.—We make this suggestion now that those who may hereafter regard their cases of sufficient moment to report, will be understood as to their judgment of such, and also that their motives in the treatment may be appreciated.

The case of Typhoid fever as reported, will be read with interest, and the treatment will doubtless be considered by those who have met similar cases in their practice. For ourselves we regard the use of mercurials on the onset as necessary, but after this we have not found them of service. In many cases, indeed, we have observed that they were contra indicated. In our treatment of this form of enteric fever we must be careful that we do not aggravate a present local difficulty and increase existing lesions, the result of which is often serious notwithstanding the greatest care and caution. Quinine, when toward the decline of the fever and when periodical chills supervene, is often valuable, but even here care and caution must be exercised otherwise cerebral disturbances may be provoked and serious lesions of the cerebral structure produced. Opium, too, must be guradedly given, otherwise irreparable mischief may be produced. If given, or when given, it should be in small and repeated doses simply to keep up sensorial power. Severe purgation in any stage we regard as an unpardonable practice and scarcely to be forgiven, and ptyalism is not to be thought of.

The truth is we are among those who do not believe in the abortive treatment of typhoid fever by any mode of proceedure. It *will* run its course and our treatment must therefore be expectant. The truth is as little treatment, or in other words, as little medication as possible is in a large majority of cases, the preferable course.

After the *prima via* have been moderately swept out on the onset, then watch the symptoms. If the pulse runs up to 100 or over then examine carefully for some local lesion. You may find some gastric difficulty presenting itself in an unusually augmented form, or there may be some exalted pulmonary lesion, the enteric symptoms may be unusually increased, or the poison may be exerting its force upon the meniences of the brain. All these should be enquired into, never forgetting the state of the skin and the urinary excretions. Cupping or blistering or both will be found valuable in local lesions, the latter particularly in the decline of the fever. We have been repeatedly gratified with the happy results of blisters to the abdomen for the local enteric difficulty and we cannot too warmly recommend a resort to these under the above named circumstances.

When the tongue is loaded there is no better alternative or organic stimulant than the emulsion of ol. turpentine. From 10 to 20 drops of the oil in mucilage given every 2, 4 or 6 hours will generally be followed by an improvement in the secretions. Its effect upon mucous surfaces is too well known to be detailed in this brief note, and therefore the propriety of its exhibition is at once apparent in these enteric fevers in which there is an almost universal lesion of the mucous lining of the bowels involving in the morbid change Peyers follicles. If the skin be dry the combination of the spirits mindereri in the proportion of two parts to one of spts. nitre dulc will generally answer for this indication as well as to promote renal depuration. The chlorate of potass in solution is also valuable as it excites the kidney to increased action. In fine, look at your patient who is laboring under typhoid fever as poisoned, to remove which, even slowly, all the emunctories must be awakened. If they are already awake do not interfere, but watch the operations of nature.—Prof. Allen had charge of a case recently which resulted favorably and in which the skin was unusually active in depurating away the morbid poison. It was not that watery exudation which is the result of a congested condition of the capillary vessels but an increased working of the sudoriparous follicles, sucking constantly and industriously the poison from the blood and throwing it away by the surface. The kidneys were also busy in the office of depuration but the bowels were a *little behind* in their work when they were gently persuaded to join in this effort of purification and redemption. The former were not interfered with in their office but left to their legitimate action. In this case had active purgation been resorted to, upon a bare presumption of necessity, there would have been a change of this momentum to structures less able to bear this increased action, and serious results would doubtless have followed. Moderate diarrhœa, when the character of the fecal discharges show that the bowels are throwiug off poison from the blood through their exhalents, is always profitable and should not be checked. At the same time invite the kidneys and the skin to unite in this work. This accomplished and there is little danger of excessive diarrhœa.

One remark more and we will close what was intended, when we set out, to be but a few brief remarks on this subject. The delirium which is often an accompanying symptom is too often supposed to arise from some lesion of the brain or its meninges. In a very large proportion of these cases this is not the true pathological condition. It generally arises from a deficiency of brain force and needs small doses of opium and also nutrition in the shape of animal jellies, Leibig's broth or chicken water in small portions repeatedly given. The repeated exhibition of nutritous food is fast becoming a favorite measure in the treatment of the low form of fevers.

The second case referred to by the Dr. was doubtless congestion of the brain and spinal cord, united there no doubt, by some previous condition of the cerebro-spinal structure. No treatment would have availed, we think, but cupping at the nape of the neck and along the course of spine, followed by powerful local stimulants, as Granville's lotion, would suggest themselves as promising something. The pulsation felt was doubtless the abdominal aorta above the bifurcation.

The Dr.'s case of surgery may appear hereafter, and, we would remark here that, as we said in the outset, the Dr.'s example should be followed and the history of every case faithfully kept. We trust he will continue his favors from time to time. Ed.

